# MN/CA IX/G250 as a potential target for immunotherapy of renal cell carcinomas

**DOI:** 10.1038/sj.bjc.6690757

**Published:** 1999-10

**Authors:** H Uemura, Y Nakagawa, K Yoshida, S Saga, K Yoshikawa, Y Hirao, E Oosterwijk

**Affiliations:** Department of Urology, Nara Medical University, 840 Shijo-cho, Kashihara, Nara, 634-8522, Japan; Second Department of Pathology, Aichi Medical University, Aichi, Japan; Department of Urology, University Hospital Nijmegen, The Netherlands

**Keywords:** renal cell carcinoma, MN/CA IX/G250 antigen, *MN/CA9/G250* gene, RT-PCR, marker

## Abstract

The monoclonal antibody G250 (mAbG250) raised against a human renal cell carcinoma (RCC) has been shown to react with a large number of RCCs. Recently, G250 antigen was isolated and found to be homologous to the *MN/CA9* gene originally identified in HeLa cells. To determine whether G250 antigen (MN/CA IX/G250) could be a potential therapeutic target and a tumour marker, a total of 147 cases of RCC were investigated immunohistochemically as well as by reverse transcriptase polymerase chain reaction (RT-PCR) analysis. In addition, total RNAs extracted from patients' peripheral blood samples were analysed for *MN/CA9/G250* mRNA signals. Immunohistochemistry demonstrated strong expression in 128/147 (87.1%) of RCCs, in contrast to the lack of expression observed in normal tissues. RT-PCR analyses of frozen specimens resulted in the clear detection of *MN/CA9/G250* mRNA signals in 137/147 (93.2%), and despite subtle differences the results were almost identical to those for immunohistochemistry. Although high-grade and -stage tumours exhibited significantly lower expression than low-grade and -stage tumours, a large proportion of tumours expressed MN/G250 protein as well as mRNA. RT-PCR analysis of patients' blood samples revealed the presence of circulating *MN/CA9/G250* expressing cells. These findings suggest that this antigen may be a potential therapeutic target as well as diagnostic marker for RCCs. © 1999 Cancer Research Campaign


*British Journal of Cancer* (1999) **81**(4), 741–746
© 1999 Cancer Research Campaign
Article no. bjoc.1999.0757

### MN/CA IX/G250 as a potential target for immunotherapy of renal cell carcinomas

**H Uemura[1], Y Nakagawa[1], K Yoshida[1], S Saga[2], K Yoshikawa[2], Y Hirao[1] and E Oosterwijk[3]**

[1]Department of Urology, Nara Medical University, 840 Shijo-cho, Kashihara, Nara 634-8522, Japan; [2]Second Department of Pathology, Aichi Medical University, Aichi, Japan; [3]Department of Urology, University Hospital Nijmegen, The Netherlands

**Summary** The monoclonal antibody G250 (mAbG250) raised against a human renal cell carcinoma (RCC) has been shown to react with a large number of RCCs. Recently, G250 antigen was isolated and found to be homologous to the *MN/CA9* gene originally identified in HeLa cells. To determine whether G250 antigen (MN/CA IX/G250) could be a potential therapeutic target and a tumour marker, a total of 147 cases of RCC were investigated immunohistochemically as well as by reverse transcriptase polymerase chain reaction (RT-PCR) analysis. In addition, total RNAs extracted from patients' peripheral blood samples were analysed for *MN/CA9/G250* mRNA signals. Immunohistochemistry demonstrated strong expression in 128/147 (87.1%) of RCCs, in contrast to the lack of expression observed in normal tissues. RT-PCR analyses of frozen specimens resulted in the clear detection of *MN/CA9/G250* mRNA signals in 137/147 (93.2%), and despite subtle differences the results were almost identical to those for immunohistochemistry. Although high-grade and -stage tumours exhibited significantly lower expression than low-grade and -stage tumours, a large proportion of tumours expressed MN/G250 protein as well as mRNA. RT-PCR analysis of patients' blood samples revealed the presence of circulating *MN/CA9/G250* expressing cells. These findings suggest that this antigen may be a potential therapeutic target as well as diagnostic marker for RCCs. © 1999 Cancer Research Campaign

**Keywords:** renal cell carcinoma; MN/CA IX/G250 antigen; *MN/CA9/G250* gene; RT-PCR; marker

Despite the fact that a variety of therapeutic strategies for urological malignancies have achieved a substantial improvement in prognoses, the renal cell carcinoma (RCC) largely remains an exception, and curative treatment of progressive RCC remains extremely difficult. It is well known that RCC displays a unique biological profile (Ritchie et al, 1987), e.g. long dormancy of metastases and spontaneous regression of metastatic disease, suggesting that RCC patients can raise abortive immune responses against as yet unknown tumour components. Investigations of RCC patient's immune status have also indicated that this influences the biological behaviour of the tumour (De Riese et al, 1991). Since RCC is relatively resistant to conventional treatment, such as radiation and chemotherapy, immunotherapy seems to be the most promising therapeutic approach. Although biological response modifiers have shown a substantial efficacy in selected cases, the response rates hardly exceed 30% and most patients cannot be cured (Wirth, 1993). Clearly, tumour-specific immunotherapy would be advantageous but unequivocal evidence for the existence of unique tumour-specific antigens in human malignancies is still lacking. A murine monoclonal antibody G250 (mAbG250) raised against human RCC is, however, known to react with a large proportion of RCCs, whereas the reactivity with normal tissues is restricted to cells of gastric mucosa and large bile ducts (Oosterwijk et al, 1986). Taking advantage of the specific reactivity with RCC, we have shown that treatment of mice bearing RCC xenografts with mAbG250 results in significant anti-tumour effects (Van Dijk et al, 1995). We also have shown that vaccination with the internal image anti-idiotype antibodies against mAbG250 results in the destruction of established RCC tumours, suggesting potential benefit with surrogate antigens for active specific immunotherapy (Uemura et al, 1994*a*, 1994*b*). Clinical trials with $^{131}$I-mAbG250 as well as $^{131}$I-chimeric mAbG250 (ch-mAbG250) for radiodiagnosis have shown safety and tumour specificity (Oosterwijk et al, 1993; Steffens et al, 1997). These results suggest that this antigen can be useful as a therapeutic and diagnostic target.

*Received 12 October 1998*
*Revised 28 April 1999*
*Accepted 29 April 1999*

*Correspondence to:* H Uemura

Recently, the G250 antigen was sequenced and was revealed by database analysis to be homologous to the MN/CA IX antigen, a tumour-associated antigen originally identified in HeLa cells (Pastorek et al, 1994; Oosterwijk et al, 1996). This antigen (MN/CA IX/G250) is a plasma membrane glycoprotein with an apparent molecular weight of 54/58 kDa, detectable in several types of malignancies; e.g. cervical and ovarian cancer (Liao et al, 1996), renal cancer (Oosterwijk et al, 1986), colorectal cancer (Saarnio et al, 1998), oesophageal cancer (Turner et al, 1997), bladder cancer (Uemura et al, 1996), but not in the normal tissues except alimentary tract, suggesting a possible role in oncogenesis. Sequential analysis has demonstrated that this gene (*MN/CA9/G250*) is a novel member of the carbonic anhydrase (*CA*) family and MN/CA IX/G250 is considered to be the only tumour-associated CA isoenzyme. Although the functional significance of MN/CA IX/G250 in oncogenesis remains unclear, MN/CA IX/G250 can induce malignant phenotype in murine NIH 3T3 fibroblasts, suggesting that MN/CA IX/G250 plays a role in control of cell proliferation and transformation (Pastorek et al, 1994). In addition, recent reports confirmed that MN/CA IX/G250 may be useful as a potentially important biomarker for cervical carcinomas (Liao et al, 1994; Brewer et al, 1996) and RCC (Liao et al, 1997; McKiernan et al, 1997). However, MN/CA IX/G250

has also been detected in normal tissues in the alimentary tract (Pastoreková et al, 1997). A sequential study of stomach cDNA showed no difference between normal and tumour tissue-derived cDNAs, indicating that *MN/CA9/G250*-associated oncogenesis may depend on the gene expression without mutations (Pastoreková et al, 1997). Moreover, a recent investigation by Ivanov et al (1998) focused on the genes involved in von Hippel–Lindau (VHL)-mediated carcinogenesis, and indicated down-regulation of *MN/CA9/G250* gene expression in RCC cell lines by wild-type *VHL* transgenes (Ivanov et al, 1998).

Thus, *MN/CA9/G250* gene may be related to carcinogenesis in RCC and MN/CA IX/G250 antigen may be a tumour-associated biomaker in RCC. Although the G250 antigen has been cloned and found to be homologous to *MN/CA9*, a precise correlation between *MN/CA9* gene and G250 antigen expression has not been shown. Therefore, we evaluated MN/G250 expression in a large number of RCC extensively by both immunohistochemistry and RT-PCR assay, and correlated the data with clinicopathological parameters. Moreover, since the haematogenous metastasis of RCC is well established and there is no reliable serological marker as a prognostic indicator, we applied RT-PCR technology for molecular detection of circulating *MN/CA9/G250*-positive cells in blood samples from RCC patients.

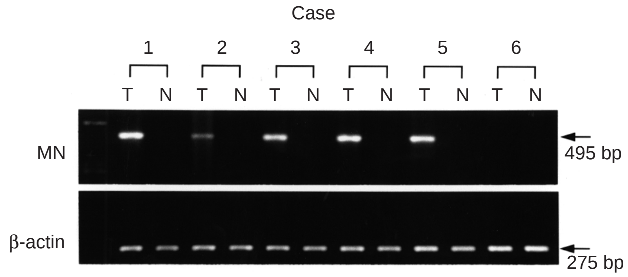

**Figure 1** *MN/CA9* assessed by RT-PCR analysis in RCC patients. Case numbers 1–5 (clear cell or granular cell type, grade 1–2 RCCs) demonstrate clear expression of *MN/CA9* mRNA whereas case number 6 (spindle cell type, grade 3 RCC) is negative; like all the corresponding normal kidney tissues. Representative results are shown

### MATERIALS AND METHODS

### Cells

Human RCC SK-RC series cell lines, reactive and non-reactive with mAbG250, were obtained from the Sloan-Kettering Institute, New York. YCR-1 was kindly supplied by Professor T Shuin of the Department of Urology, Kochi Medical College. Cells were cultured in RPMI-1640 medium supplemented with L-glutamine and 10% fetal bovine serum.

### Specimens

RCC and corresponding normal kidney tissues were obtained by radical operations in our department. All specimens were certainly kept under RNAase-free conditions immediately after nephrectomy, and small pieces of tumour or normal kidney tissue were frozen in liquid nitrogen and stored at –80°C. The remainder of each kidney was submitted for pathological examination by the central pathologist (KI) at this institute.

### Monoclonal antibody

A murine monoclonal antibody, mAbG250 (IgG1, IgG2a), which recognizes the MN/CA IX/G250 antigen, was used for immunohistochemical staining and mixed haemadsorption assays (MHA). The isolation and characterization of mAbG250 has been described elsewhere (Oosterwijk et al, 1986).

### Immunohistochemistry of RCC specimens

Immunohistochemistry of frozen tissues was carried out as described previously (Uemura et al, 1995). Briefly, 4–5-micron-thick cryostat sections were airdried for 1 h and fixed in acetone/methanol for 10 min at room temperature. After washing in phosphate-buffered saline (PBS), tissue sections were incubated with mAbG250 for 1 h at room temperature. After a three further washes with PBS, the tissue sections were incubated with goat anti-mouse IgG conjugated to horseradish peroxidase, preincubated with 10% normal human serum. The tissue sections were developed with 3-3′-diaminobenzidine and 0.01% hydrogen peroxide for 10 min at room temperature. After washing with tap water, the slides were counterstained with haematoxylin, and mounted with Permount.

### Mixed haemadsorption assay

RCC cell lines were examined for MN/CA IX/G250 antigen expression on the cell surface by MHA. Briefly, the cells cultured in 60-well plates overnight were incubated with appropriately diluted mAbG250 for 60 min at room temperature, and subsequently with indicator sheep red blood cells (SRBC) treated with mouse anti-SRBC and goat anti-mouse IgG antibody for 60 min at room temperature. The reaction was determined as positive when over 25% of the target cells formed rosettes.

### RT-PCR analysis

Peripheral blood lymphocytes separated by centrifugation using Ficoll-Conray (IBL, Gunma, Japan), tissues and cell lines were used for preparation of total RNAs by homogenization in guanidium thiocyanate and centrifugation through caesium chloride. Total RNAs were reverse transcribed with SuperScript RNAase H reverse transcriptase (Gibco-BRL), using random primers. The resulting cDNA fragments were then amplified by PCR, using combined sense and anti-sense degenerate primers (sense primer; 5′-ACTGCTGCTTCTGATGCCTGT-3′, 126–146 nt, anti-sense; 5′-AGTTCTGGGAGCGGCGGGA-3′, 620–602 nt). The amplification was achieved with 30 cycles, each consisting of denaturation (95°C, 2 min), annealing (68°C 2 min), and extension (72°C, 1 min). The PCR products from several tissue RNAs were 495 bp in length. For blood samples, to obtain a higher sensitivity of the RT-PCR assay, PCR Southern blots were carried out. Briefly, PCR products were electrophoresed in 1.5% agarose gels and transferred to nitrocellulose filters. The cDNA probe (167–447 nt) was hybridized with filters at 42°C in the presence of 40% formamide, and the filters were finally washed with 0.2 × standard saline citrate, 0.1% sodium dodecyl sulphate at 60°C. The size of the DNA was determined by comparison with lambda phage DNA fragments digested with *Hin*dIII.

 *© 1999 Cancer Research Campaign*

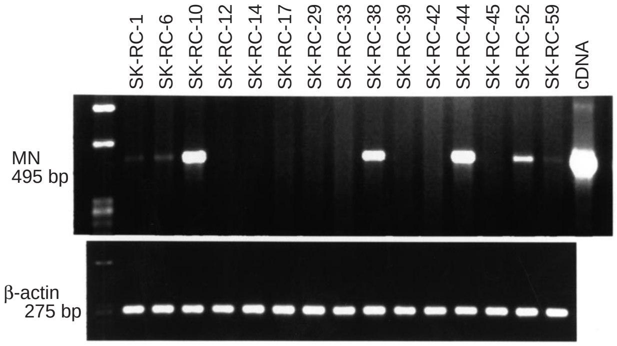

**Figure 2** Results of RT-PCR analysis of MN/CA9 in RCC cell lines. Seventeen RCC cell lines, SKRC-1, 6, 10, 12, 14, 17, 29, 33, 38, 39, 42, 44, 45, 52, 59, ACHN and YCR-1 were analysed by the RT-PCR method. G250 cDNA was used as a positive control. Representative results are shown

**Table 1** MN/G250 expression with respect to tumour stage and grade

| | Stage | | | | Total | |
|---|---|---|---|---|---|---|
| | $T_1$ | $T_2$ | $T_3$ | $T_4$ | | |
| MN/G250+ | 25 | 68 | 32 | 3 | 128 (87.1%) | $P < 0.005$ |
| MN/G250– | 2 | 3 | 10 | 4 | 19 (12.9%) | |
| | **Grade** | | | | **Total** | |
| | **G1** | **G2** | **G3** | | | |
| MN/G250+ | 69 | 54 | 5 | | 128 (87.1%) | $P < 0.0001$ |
| MN/G250– | 1 | 8 | 10 | | 19 (12.9%) | |

### RT-PCR assay sensitivity for detection of circulating *MN/CA9/G250*-positive cells

To determine the sensitivity of the RT-PCR assay for detection of circulating *MN/CA9/G250*-expressing cells, SKRC-44 (*MN/CA9/G250*-positive cell line) cells were serially diluted into 10 ml whole blood obtained from a 36-year-old healthy male volunteer (*MN/CA9/G250*-negative blood). Briefly, a single cell suspension of SKRC-44 was prepared in PBS and serial dilutions were carried out. Different dilutions were added to 10-ml aliquots of normal blood samples, to give concentrations of 0, $10^0$, $10^1$, $10^2$, $10^3$, $10^4$, $10^5$, $10^6$ cells per 1 ml whole blood. For lymphocyte separation, the blood samples were centrifuged using Ficoll–Conray. Total RNA was extracted from the cell pellets and RT-PCR analysis was performed as described above.

### Statistical analysis

Correlations between MN/CA IX/G250 expression and clinico-pathological parameters were evaluated using the $\chi^2$ test. A probability < 0.05 was considered significant.

### RESULTS

### MN/G250 expression in RCC specimens mAbG250 stained 128 of 147 (87.1%) cases, most demonstrating homogeneous and strong binding. The staining strength and percentages of positive tumour cells correlated in different areas of the same RCC, in accordance with the results of our previous study. Data for relations between MN/CA IX/G250 expression and tumour stage and grade are summarized in Table 1. Significant inverse links were noted in both cases $P < 0.005$ and $P < 0.0001$ respectively, i.e. high-grade tumours expressed MN/CA IX/G250 antigen at a significantly lower rate (33.3%) than low-grade tumours (98.6%). MN/CA IX/G250 expression of tumour cells was not related to the morphological type of RCC (Tables 2 and 3), i.e. both clear cell and non-clear cell types expressed MN/CA IX/G250. However, the proportion of clear cell tumours (95/96, 99.0%) homogeneously stained was greater than for the non-clear cell type (33/51, 64.7%). RT-PCR analyses of RCC frozen specimens resulted in clear detection of *MN/CA9/G250* mRNA signals (137/147, 93.2%) (Figure 1). However, the results were not identical to those of immunohistochemical analysis (Tables 2 and 3). All positive stained tumours with mAbG250 expressed *MN/CA9/G250* mRNA by RT-PCR, but not vice versa, i.e. nine tumours expressed *MN/CA9/G250* mRNA by RT-PCR but did not demonstrate MN/CA IX/G250 protein on immunohistochemistry.

### MN/G250 expression in RCC cell lines

In eight of 17 RCC cell lines (47.0%), *MN/CA9/G250* mRNA signals were observed by RT-PCR analysis (Figure 2), and of these eight RT-PCR(+) cell lines, only five expressed MN/CA IX/G250 antigen by MHA (Table 4). All MHA-positive cell lines (SKRC-6, 10, 38, 44, 52) expressed MN/CA9 mRNA, but not vice versa.

### Detection of circulating *MN/CA9/G250*-positive cells in the blood

RT-PCR sensitivity assay revealed that one single *MN/CA9/G250*-positive cell in 1 ml whole blood was detectable (Figure 3). Standard RT-PCR analysis (30 cycles) revealed the presence of circulating *MN/CA9/G250*-positive cells in several blood samples taken from inferior vena cava or renal vein during radical nephrectomies, but not in peripheral blood samples (data not shown). To obtain high sensitivity for detecting *MN/CA9/G250*-positive circulating cells, nested PCR as well as PCR Southern blotting analyses were carried out (Figure 4). Thereby, 76.2% (32/42) of blood samples from RCC patients demonstrated *MN/CA9/G250* mRNA, although this was also the case for 32.2% (10/31) of the blood samples from healthy donors without any disease (Table 5 and Figure 4). The presence of *MN/CA9/G250*-expressing cells in control blood was confirmed by the sequence analysis (data not shown).

### DISCUSSION

RCC is relatively resistant to conventional anticancer treatment such as radiation, chemotherapy or hormonal therapy, and radical operations are considered the only way to achieve cures. However, it also has a unique biological profile, demonstrating long dormancy of metastasis and spontaneous regression after nephrectomy, and an investigation concerning immune status of patients with RCCs revealed that the biological behaviour of tumours was influenced by host factors (Ritchie et al, 1987). So far, immunotherapy seems to be the most promising treatment approach and cytokine immunotherapy for RCC, e.g. with interferons and interleukin-2, has worldwide

© 1999 Cancer Research Campaign

**Table 2** MN/CA9/G250 expression with respect to cell type

| | No. of cases | Positive cases (%) | |
|---|---|---|---|
| | | mAbG250 (%) | RT-PCR (%) |
| Clear cell | 96 | 95 (99.0) | 96 (100) |
| Granular cell | 29 | 19 (65.5) | 23 (79.3) |
| Mixed cell | 17 | 13 (76.5) | 17 (100) |
| Spindle cell | 2 | 0 (0.0) | 0 (0.0) |
| Pleomorphic cell | 3 | 1 (33.3) | 1 (33.3) |
| | 147 | 128 (87.1) | 137 (93.2) |

**Table 3** MN/CA9/G250 expression with respect to growth pattern

| | No. of cases | Positive cases (%) | |
|---|---|---|---|
| | | mAbG250 (%) | RT-PCR (%) |
| Alveolar | 106 | 101 (95.3) | 105 (99.1) |
| Tubular | 17 | 16 (94.1) | 16 (94.1) |
| Papillary | 7 | 3 (42.9) | 4 (57.1) |
| Cystic | 5 | 3 (60.0) | 3 (60.0) |
| Solid | 7 | 1 (14.3) | 4 (57.2) |
| Mixed | 5 | 4 (80.0) | 5 (100) |
| | 147 | 128 (87.1) | 137 (93.2) |

**Table 4** MN/G250 expression in RCC cell lines

| Cell line | | RT-PCR | MHA |
|---|---|---|---|
| SKRC | –1 | + | – |
| | –6 | + | + |
| | –10 | + | + |
| | –12 | – | – |
| | –14 | – | – |
| | –17 | – | – |
| | –29 | – | – |
| | –33 | – | – |
| | –38 | + | + |
| | –39 | – | – |
| | –42 | – | – |
| | –44 | + | + |
| | –45 | – | – |
| | –52 | + | + |
| | –59 | + | – |
| ACHN | | – | – |
| YCR-1 | | + | – |

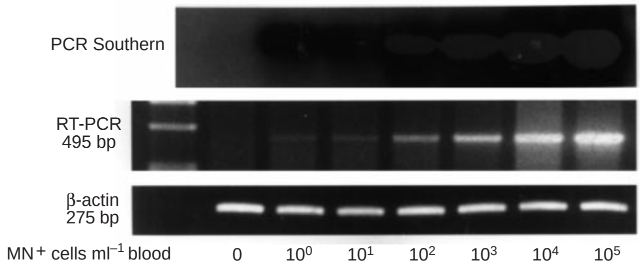

**Figure 3** Sensitivity assay of RT-PCR and PCR Southern blot. Different dilutions of SKRC-44 (MN/CA IX/G250-positive) cell suspensions were added to 10 ml aliquots of normal whole blood. Until at concentration of one SKRC-44 cell in 1 ml whole blood, the mRNA signal can be detectable

**Table 5** Detection of circulating MN/G250-positive cell

| | MN/G250+ | MN/G250– | Total |
|---|---|---|---|
| RCC | 33 (76.7%) | 10 (23.3%) | 43 |
| Normal | 10 (32.3%) | 21 (67.7%) | 31 |

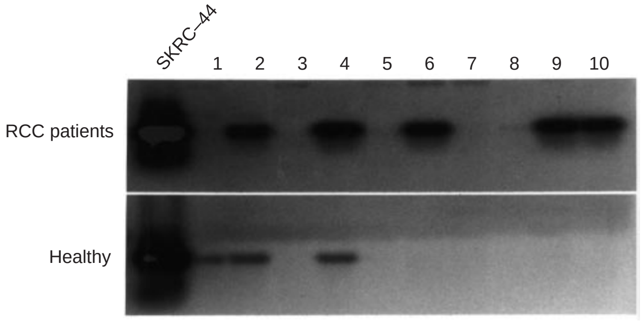

**Figure 4** MN/CA9 expression in blood samples, as analysed by PCR Southern blotting. Blood samples from 43 RCC patients as well as 31 healthy volunteers were investigated. RNA obtained from the SKRC-44 RCC cell line was used as a positive control. Representative results are shown become the most common first therapeutic alternative next to nephrectomy. Nevertheless, the prognosis of RCC patients with disseminated disease is still extremely poor, and a more specific therapeutic approach is needed. We have focused on the G250 antigen expressed on a large proportion of RCCs as a promising target molecule. Isolation and sequential analysis revealed G250 to be identical to MN/CA IX (Oosterwijk et al, 1996), a N-glycosylated protein originally identified in HeLa cells by mAb M75 (Pastoreková et al, 1992) and distributed on the plasma membrane as well as in the nucleus. Immunohistochemical studies with mAb M75 as well as mAbG250 for MN/CA IX/G250 expression have been carried out in several malignancies. In RCC, the first immunohistochemical study of MN/CA IX/G250 was reported by Oosterwijk et al (1986) using mAbG250. However, it was not considered that both mAb M75 and mAbG250 recognized the same antigen MN/CA IX/G250. Another report by Liao et al (1997) using mAb M75 demonstrated very similar results, i.e. 45 of 47 primary RCC (95%) were MN/CA IX/G250-positive, all clear cell tumours with or without granular/spindle cell expressing MN/CA IX/G250. Our extensive study with mAbG250 revealed an inverse correlation between MN/CA IX/G250 expression and tumour stage and grade, as described in other diseases (Brewer et al, 1996; Turner et al, 1997; Saarnio et al, 1998). Brewer et al (1996) have shown that low expression of MN/CA IX/G250 correlates with poor prognostic factors, e.g. poorly differentiated tumour, lymph node metastasis, depth of cervical stromal invasion, suggesting low MN/CA IX/G250 expression in tumour cells with higher malignant potential. Similar results have been reported in colorectal cancer (Saarnio et al, 1998) and oesophageal cancer (Turner et al, 1997). After cloning of *MN/CA9* gene, this gene expression of benign human tissues and RCC was investigated by RT-PCR assay using an oligonucleotide primer specific for *MN/CA9/G250* cDNA (McKiernan et al, 1997). In this RT-PCR study of 17 RCC cases, 12 pure clear cell type tumours

 *© 1999 Cancer Research Campaign*

showed *MN/CA9/G250* expression and no expression was observed in the remaining five granular or papillary cell type tumours with or without clear cell elements. Although the first report by Oosterwijk et al (1996) emphasized that reactivity with mAbG250 is not related to morphological appearance of the cells, the two current reports suggest that MN/CA IX/G250 may be cell-specific tumour-associated protein, i.e. a useful potential marker for clear cell type RCC.

Based on our results, a majority of RCC are MN/CA IX/G250-positive; mAbG250(+)/RT-PCR(+) in 128 (87.1%), mAbG250(–)/RT-PCR(+) in nine (6.1%) and mAbG250(–)/RT-PCR(–) in ten cases (6.8%), respectively, with RT-PCR being a most reliable detection method. It seems that MN/CA IX/G250 expression is related to the clear cell type, with alveolar or tubular structures. However, some non-clear cell type tumours with or without clear cell elements were also MN/CA IX/G250-positive. Immunohistochemical and RT-PCR analyses for these groups were consistent, so they may be indicated for immunotherapy. With the others, where the RT-PCR approach was more sensitive, diagnostic applications may still be expected. Despite the fact that the standard RT-PCR (30 cycles) resulted in clear detection of *MN/CA9/G250* mRNA, nine RCC specimens and two cell lines did not show MN/CA IX/G250 antigen expression. This discrepancy may be due to lower sensitivity of immunohistochemical analysis. With respect to the clinical usefulness of MN/CA IX/G250 antigen as a target molecule, 19 (12.9%) of the mAbG250(–) cases might be categorized as negative.

To further investigate whether *MN/CA9/G250* could be a potential diagnostic marker, the RT-PCR assay was used to detect circulating *MN/CA9/G250*-positive cells in blood from RCC patients. Approximately three-quarters of the blood samples showed clear expression of *MN/CA9/G250* mRNA, whereas only one-third of healthy donor samples were positive. Sequence analysis confirmed the presence of circulating *MN/CA9/G250*-positive cells in the control blood. With regard to the question of where these *MN/CA9/G250* antigen-positive cells come from, we are currently investigating a variety of normal tissues including haematogenous organs and haematopoietic cell lines, but so far, no clear expression of *MN/CA9/G250* has been observed except in the alimentary tract (data not shown). McKiernan et al checked MN/CA9 expression of several normal tissues including normal peripheral blood samples by RT-PCR with different primers and showed no expression in any normal samples (McKiernan et al, 1997). However, they subsequently also found *MN/CA9/G250*-expressing cells in normal blood samples (McKiernan et al, 1998). This was confirmed by our RT-PCR analysis with the same primers and conditions as they employed (data not shown). Similar results have been reported for other malignancies. For example, wide ranges of antigen detection rates of circulating prostatic-specific antigen (PSA) or prostatic-specific membrane antigen (PSM) positive cells in prostate cancer cases have also been described (Hamdy et al, 1992; Sokoloff et al, 1996; Ignatoff et al, 1997). With the RT-PCR analyses of MacKiernan and ourselves, hypersensitive RT-PCR assays with PCR Southern blots or nested RT-PCR (total 50 cycles), allowed detection of *MN/CA9/G250*-expressing cells in peripheral blood samples. To confirm whether circulating *MN/CA9/G250*-positive cells could be real RCC cells, further investigation with additional markers is required. Unfortunately, there is no reliable marker specific for RCC detection presently available. Since normal alimentary tissues express MN/CA IX/G250, further precise study with other normal tissues including haematological cells, is needed to investigate the possible significance of MN/CA IX/G250-positive cells in normal blood samples. However, because of the significantly high detection rate in RCC, quantitative analysis of *MN/CA9/G250* expression might allow discrimination from non-RCC. In order to obtain both high sensitivity and specificity for detection of circulating *MN/CA9/G250*-positive cells, optimal settings of RT-PCR are under investigation.

In conclusion, our investigation of both MN/CA IX/G250 and *MN/CA9/G250* gene expression in a large number of RCC specimens revealed the majority to express MN/CA IX/G250. Immunohistochemical findings with mAbG250 (128/147, 87.1%) was almost identical to the results of RT-PCR analysis (137/147, 93.2%). We also showed that RT-PCR analysis of blood samples from RCC patients can detect circulating *MN/CA9/G250*-positive cells, although the sensitivity (32/42, 76.2%) and specificity (21/31, 77.8%) were relatively low. Our findings suggest that the *MN/CA9/G250* antigen is a promising tool as a therapeutic target and a diagnostic marker for detection of residual disease after RCC resection.

### ACKNOWLEDGEMENTS

The authors wish to thank Dr K Ichijima for detailed pathological diagnosis of RCC specimens. This work was partly supported by Grants-in-Aid for Scientific Research (C) (08671834 and 09671643) from the Ministry of Education, Science, Sports and Culture, Japan.

### REFERENCES

Allhoff EP, Liedke S, Kirchner H, Atzpodien J, De Riese W, Stief CG and Jonas U (1991) Current clinical relevance of immunotherapy in metastatic renal cancer. *World J Urol* **9**: 228–231

Brewer C, Liao SY, Wilczynski SP, Pastrekova S, Pastorek J, Zavada J, Kurozaki T, Manetta A, Berman ML, DiSaia PJ and Stanbridge EJ (1996) A study of biomarkers in cervical carcinoma and clinical correlation of the novel biomarker MN. *Gynecol Oncol* **63**: 337–344

De Riese W, Allhoff EP, Kirchner H, Stief CG, Atzpodien J, Maschek H and Jonas U (1991) Complete spontaneous regression in metastatic renal cell carcinoma – an update and review. *World J Urol* **9**: 184–191

Hamdy FC, Lawry J, Anderson JB, Parsons MA, Rees RC and Williams JL (1992) Circulating prostate specific antigen-positive cells correlate with metastatic prostate cancer. *Brit J Cancer* **69**: 392–396

Ignatoff JM, Oefelein MG, Watkin W, Chmiel JS and Kaul KL (1997) Prostate specific antigen reverse transcriptase-polymerase chain reaction assay in preoperative staging of prostate cancer. *J Urol* **158**: 1870–1875

Ivanov SV, Kuzmin I, Wei MH, Pack S, Geil L, Johnson BE, Stanbridge EJ and Lerman MI (1998) Down-regulation of transmembrane carbonic anhydrases in renal call carcinoma cell lines by wild-type von Hippel-Lindau transgenes. *Proc Natl Acad Sci USA* **95**: 12596–12601

McKiernan JM, Buttyan R, Bander NH, Stifelman MD, Katz AE, Chen MW, Olsson CA and Sawczuk IS (1997) Expression of the tumor-associated gene MN: a potential biomarker for human renal cell carcinoma. *Cancer Res* **57**: 2362–2365

McKiernan JM, Stifelman MD, Emanuel ER, Katz AE, Bander NH, Olsson CA, Buttyan R and Sawczuk IS (1998) The molecular detection of renal cancer cells via a peripheral blood assay. *J Urol Suppl* **159**: 170

Liao SY, Brewer C, Zavada J, Pastorek J, Pastreková S, Manetta A, Berman ML, DiSaia PJ and Stanbridge EJ (1994) Identification of the MN antigen as a diagnostic biomarker of cervical intraepithelial squamous and glandular neoplasia and cervical carcinomas. *Am J Pathol* **145**: 598–609

Liao SY, Aurelio ON, Jan K, Zavada J and Stanbridge EJ (1997) Identification of the MN/CA9 protein as a reliable diagnostic biomarker of clear cell carcinoma of the kidney. *Cancer Res* **57**: 2827–2831

Oosterwijk E, Ruiter DJ, Hoedemaeker JC, Huiskens-V JW, Meij D, Jonas U, Fleuren GJ, Zwartendijk J, Hoedemaeker PJ and Warnaar SO (1986) Immunohistochemical analysis of monoclonal antibodies to renal antigens. *Am J Pathol* **123**: 301–309

© 1999 Cancer Research Campaign 

Oosterwijk E, Bander NH, Divgi CR, Wakka JC, Finn RD, Carswell EA, Larson SM, Warnaar SO, Fleuren GJ, Oettgen HF and Old L (1993) Antibody localization in human renal cell carcinoma: a phase I study of monoclonal antibody G250. *J Clin Oncol* **11**: 738–750

Oosterwijk E, De Weijert M, Van Bokhoven A, Brakenhoff RH, Peelen WP and Debruyne FMJ (1996) Molecular characterization of the renal cell carcinoma-associated antigen G250. *Proc Amer Assoc Cancer Res* **37**: 461

Opavsky R, Pastoreková S, Zelník V, Gibadulinová A, Stanbridge EJ, Závada J, Kettmann R and Pastorek J (1996) Human MN/CA9 gene, a novel member of the carbonic anhydrase family: structure and exon to protein domain relationships. *Genomics* **33**: 480–487

Pastorek J, Pastoreková S, Callebaut I, Mornon JP, Zelník V, Opavsky R, Zat'ovicová M, Liao S, Portetelle D, Stanbridge EJ, Závada J, Burny A and Kettmann R (1994) Cloning and characterization of MN, a human tumor-associated protein with a domain homologous to carbonic anhydrase and a putative helix-loop-helix DNA binding segment. *Oncogene* **9**: 2877–2888

Pastoreková S, Závadoá Z, Kostál M, Babusíková O and Závada J (1992) A novel quasi-viral agent, TaTu is a two-component system. *Virology* **187**: 22–28

Pastoreková S, Parkkila S, Parkkila AK, Opavsky R, Zelník V, Saarnio J and Pastorek J (1997) Carbonic anhydrase IX, MN/CA IX: analysis of stomach complementary DNA sequence and expression in human and rat alimentary tracts. *Gastroenterology* **112**: 398–408

Ritchie AWS and deKernion JB (1987) The natural history and clinical features of renal carcinoma. *Semin Nephrol* **7**: 131–139

Saarnio J, Parkkila S, Parkkila AK, Haukipuro K, Pastoreková S, Pastorek J, Kairaluoma MI and Karttunen TJ (1997) Immunohistochemical study of colorectal tumors for expression of a novel transmembrane carbonic anhydrase, MN/CA IX, with potential value as a marker of cell proliferation. *Am J Pathol* **153**: 279–285

Sokoloff MH, Tso C-L, Kaboo R, Nelson S, Ko J, Dorey F, Figlin RA, Pang S, DeKernion J and Belldegrun A (1996) Quantitative polymerase chain reaction does not improve preoperative prostate cancer staging: a clinicopathological molecular analysis of 121 patients. *J Urol* **156**: 1560–1566

Steffens MG, Boerman OC, Oosterwijk-Wakka JC, Oosterhof GO, Witjes JA, Koenders EB, Oyen WJ, Buijd WC, Debruyne FMJ, Corstens FH and Oosterwijk E (1997) Targeting of renal cell carcinoma with iodine-131-labeled chimeric monoclonal antibody G250. *J Clin Oncol* **15**: 1529–1537

Tuner JR, Odze RD, Crum FCP and Resnick MB (1997) MN antigen expression in normal, preneoplastic, and neoplastic esophagus: a clinicopathological study of a new cancer-associated biomarker. *Human Pathol* **28**: 740–744

Uemura H, Okajima E, Debruyne FMJ and Oosterwijk E (1994*a*) Internal image antidiotype antibodies related to renal cell carcinoma-associated antigen G250. *Int J Cancer* **56**: 609–614

Uemura H, Beniers AMJC, Okajima E, Debruyne FMJ and Oosterwijk E (1994*b*) Vaccination with anti-idiotype antibodies mimicking a renal cell carcinoma-associated antigen induces tumor immunity. *Int J Cancer* **58**: 555–561

Uemura H, Okajima E, Debruyne FMJ and Oosterwijk E (1995) Anti-tumor effects induced by vaccination with anti-idiotype antibodies in 7 mice with established human renal cell carcinoma xenografts. *Urol Oncol* **1**: 73–79

Uemura H, Kitagawa H, Hirao Y, Okajima E, Debruyne FMJ and Oosterwijk E (1997) Expression of tumor-associated antigen MN/G250 in urologic carcinoma: potential therapeutic target. *J Urol Suppl* **157**: 377

Van Dijk J, Uemura H, Beniers AMJC, Peelen WP, Zegveld STh, Fleuren GJ, Warnaar SO and Oosterwijk E (1994) Therapeutic effects of monoclonal antibody G250, interferons and tumor necrosis factor, in mice with renal cell carcinoma xenografts. *Int J Cancer* **56**: 262–268

Wirth MP (1993) Immunotherapy for metastatic renal cell carcinoma. *Urol Clin N Am* **20**: 283–295

 *© 1999 Cancer Research Campaign*
